# Methods of Precise Distance Measurements for Laser Rangefinders with Digital Acquisition of Signals

**DOI:** 10.3390/s21196426

**Published:** 2021-09-26

**Authors:** Michał Muzal, Marek Zygmunt, Piotr Knysak, Tadeusz Drozd, Marcin Jakubaszek

**Affiliations:** Institute of Optoelectronics, Military University of Technology, 2 S. Kaliskiego Street, 00-908 Warsaw, Poland; marek.zygmunt@wat.edu.pl (M.Z.); piotr.knysak@wat.edu.pl (P.K.); tadeusz.drozd@wat.edu.pl (T.D.); marcin.jakubaszek@wat.edu.pl (M.J.)

**Keywords:** laser rangefinder, long range distance measurements, full waveform LiDAR, precise distance measurement algorithms

## Abstract

The article presents methods of long range distance measurements using pulsed lasers and the Time of Flight principle. Various algorithms of laser distance measurements with digital acquisition of echo pulses (acquisition of a signal’s full waveform) are presented. The main focus of work is concentrated on the method of distance measurements developed by the authors. With this method, during laboratory trials, a total measurement error of one centimeter was achieved using a 905 nm pulsed laser diode and pulse width of 39 ns. The maximum range of measurements with such high precision is limited only by a signal to noise ratio, duration of measurements and atmospheric conditions. All algorithms were implemented in a laser rangefinder module developed by the authors. Simulations and laboratory experiments were conducted and algorithm’s accuracy and precision were tested for various SNR conditions and changing distances.

## 1. Introduction

The present work is connected with an effort to develop a method of remote distance measurements that is characterized by an uncertainty that is low enough to allow remote measurements of the speed of vehicles with the total error no greater than 1 km/h. This requirement results from the Polish legal regulations relating to laser devices used in law enforcement [1]. It was also assumed that the maximum range of measurements has to be grater then 600 m, and that measurements of the speed of vehicles that are not equipped with retroreflective license plate were possible. It means measuring to any surface of the vehicle (windshield, painted surfaces, plastic parts, etc.). As a result of those requirements, we have decided to develop a measuring device that uses pulsed laser as a radiation source and performs acquisition of the full waveform of returning echo signals. Because laser measurements of vehicle speed are based on measurements of its displacement in a given time, the simplest way to do them is to measure distance to the target twice (or usually many more times) in a set time interval. Precision and accuracy of such measurement of speed strongly depend on the precision and accuracy of measurements of each distance. Through basic calculations, it is easy to derive that the required uncertainty of measurements of distance should fall in the range of centimeters when total duration of measurement of the speed doesn’t exceed half a second [1,2]. Beside two mentioned parameters—precision and accuracy—another crucial parameter of distance measurement is its maximum range. All three parameters depend on the chosen measurement method, among which exist methods based on measurements of the phase shift between transmitted and received FMCW signals (Indirect ToF) [3,4] and, whenever longer ranges are needed, methods based on measurements of the probing light pulse roundtrip Time of Flight to the target (Direct ToF) [5,6,7]. In the presented work, the pulsed Time of Flight method was chosen to allow long range measurements. 

In order to be able to safely use the designed device on public roads, we had to comply with eye safety regulations defined in adequate standards [8]. To do so, we have chosen a laser diode working at 905 nm in the class 1 limits of accessible laser emission. For such conditions, basic ToF methods that use direct detection (DD) of incoming echo signals have ranges limited to less than 200 m (when target reflectivity is close to 10%). In order to extend the range of measurements beyond the limit of direct detection, we have decided to use multi pulse coherent addition of signals (aka. multi-pulse coherent superposition) [9,10]. This required taking a series of measurements and synchronously adding recorded waveforms together in order to reduce noise. Beside noise reduction, coherent addition also significantly reduces the influence of asynchronous interferences. This is a desirable feature in police speed guns, as cars may have jamming devices installed by owners that easily interfere with measurements done by direct detection devices. It also makes measurements immune to interferences caused by other devices that emit infrared pulses such as other measurement devices or lidars on autonomous cars. Implementation of coherent addition method requires analog to digital conversion of returning signals, and the use of ADC converters is inevitably associated with loss of continuity of recorded signals due to quantization and discretization. This introduces extra noise in the signal and - without additional countermeasures - affects precision of a rangefinder with full waveform acquisition of signals (also called “full waveform LiDAR”). In the presented work we were using an ADC converter that was chosen optimally in terms of its cost, design simplicity and its sampling rate and was suitable for use in handheld laser speed guns. Because of the discretization error introduced by the chosen ADC, which was greater than the allowable total measurement error, we had to recreate signal’s continuity in both domain and codomain to precisely measure ToF. In this article, we present the method of accurate and precise determination of distance that was developed by us and compare it in simulations and laboratory experiments with simpler methods to assess its effectiveness. In the following sections of this article, selected analogue and digital methods of generation of the START and STOP signals are presented. In Section 3, we will discuss the proposed algorithm of measurements and analyze its precision. In Section 4 results of simulations, and in Section 5 results of laboratory experiments, are shown. 

## 2. Methods of Generation of the START and STOP Signals for Digital Counters in Laser ToF Measurements

As mentioned earlier, correct measurement of distance, by means of measuring the Time of Flight of light pulses, require precise generation of START and STOP signals that are used to control timers. The timing point (moment) when generation of START and STOP signals should occur is a result of observation of different characteristic points on the waveform of received signals [6]. There are many measurement methods [9,11,12] to perform this task that can be classified into three main groups that derive the moment of generation of STOP signals from:

(a) Peak Detection,
(b) Constant Threshold Detection,
(c) Adaptive Threshold Detection.

**Figure 1:**
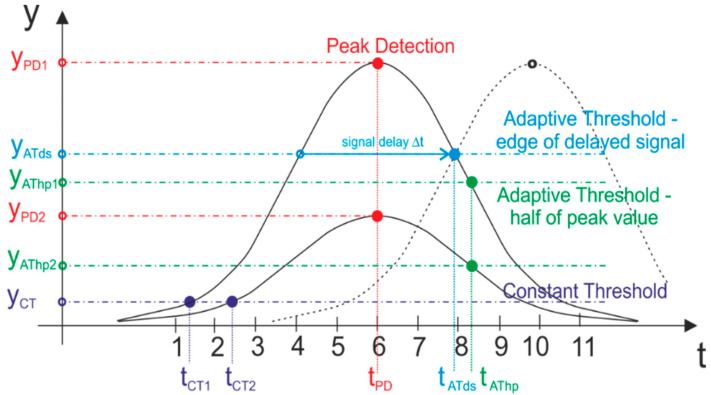
Characteristic points of the waveform of Gaussian curve modeling the echo signal of a laser pulse and names of methods based on their detection.

The Constant Threshold Detection (also called “leading edge discrimination” [11]) method is used in the simplest hardware implementations of direct ToF measurements. In this method, generation of START and STOP signals is based on comparison of the voltage of signal coming from the receiver with constant reference voltage (threshold). The main disadvantage of constant threshold detection is that the moment of generation of STOP is dependent from changes in the power of received pulse (see *t_CT1_* and *t_CT2_* in Figure 1). The purpose of more complex measurement methods is to ensure the independence of STOP signal generation time from those changes. This is a prerequisite for high measurement accuracy and elimination of the so-called walk error [7]—the dependence of measurement results from changes of peak power of received signals. This independence can be achieved, i.e., by linking generation of STOP signals with the time when peak voltage is detected (Peak Detection) or by the use of a comparator with an adaptive (variable) threshold. For analog signals, the peak detection can be implemented using a differentiating circuit and zero crossing detection of signals (detection of zero of the first derivative of a signal). The circuit implementing this method is called a high-pass timing discriminator [5]. The Adaptive Threshold is usually set to follow the changing value of the signal’s peak voltage. It can be implemented using a voltage divider that is constantly setting the reference (threshold) voltage to a fraction (i.e., half) of the peak voltage of received pulses. It can also be implemented using delay lines to delay the incoming pulse (usually, by the value of its full width at half maximum) and compare it with the original signal (see Figure 1—Adaptive Threshold—edge of delayed signal). The Adaptive Threshold method is also called “constant fraction discrimination” [11,13,14]). Figure 1 shows the characteristic features of the modeled waveform of a laser pulse signal along with names of methods that base on detection of their appearance. 

**Figure 2:**
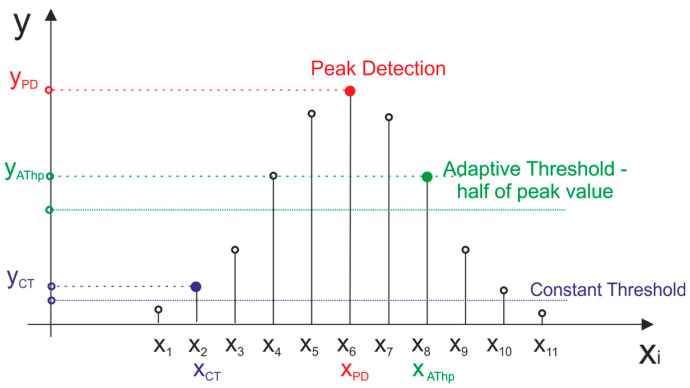
Standard detection methods applied to a digital signal.

Above methods can also be used for echo signals recorded in a digital form; however, the discontinuity of digital signals in the time domain and quantization errors can have a significant impact on the accuracy of measurements. In Figure 2, by analogy with Figure 1, the characteristic points of presented detection methods, that were applied to a digital signal, are shown. Similarly to the analog domain, in the digital domain, the Constant Threshold is implemented through searching of the position of the firs sample that has value greater or equal to the chosen threshold value. Peak Detection is implemented through searching of a sample that has the largest value in the recorded signal. Heaving the value of a peak sample of the signal opens the possibility to implement Adaptive Threshold method by simply setting the detection threshold to its chosen fraction.

**Figure 3:**
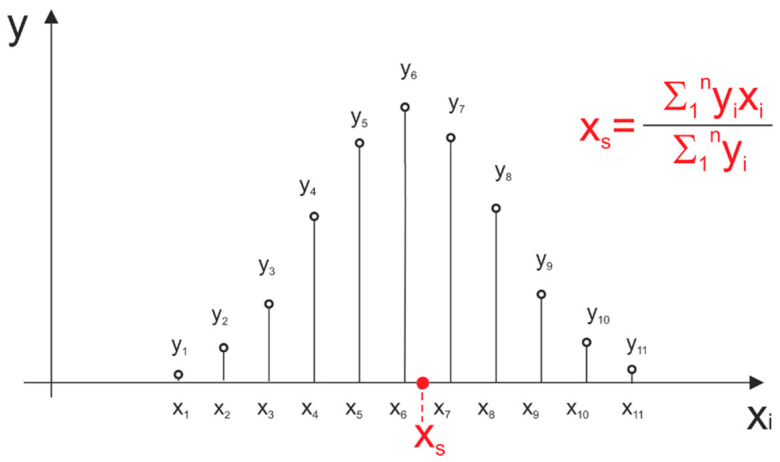
Weighted average as a method of finding the center x_s_ coordinate of digitally recorded pulse.

Obtaining precise time measurements with described methods requires either high-frequency sampling or generation of very short probing pulses, which in both cases increases the complexity of transceiver elements of the device. Typical semiconductor lasers, suitable for the discussed methods, are able to emit light pulses with a duration of a few to several dozen nanoseconds, so their detection requires use of broadband optical receivers. Shortening of the width of a probing pulse is associated with the need of a system bandwidth extension. Unfortunately, by doing so, we increase the receiver’s noise level, its sensitivity to interferences and the complexity of the electronic circuitry (the need to implement fast analog to digital converters). Thus, the effective use of an analog to digital conversion of signals in laser rangefinders calls for the development and use of measurement algorithms that differ from the ones presented so far. One of the ways of handling digital signals is to restore their continuity by chosen approximation or interpolation algorithms [6]. This approach is at the heart of the least square approximation with a second degree polynomial (SDPA) that is the main subject of this article and will be presented in Section 3. Beside using approximation, increase in the precision of measurements (Peak Detection) is possible by using signal samples to calculate the weighted average (WA) of their x coordinates. This method is presented in Figure 3.

The result of such calculations is an *x_s_* coordinate of the pulse’s “center of gravity” that approximates the position of the real peak of the signal. The weighted average of signal samples is one of methods that were used as a comparative method during the research. 

## 3. Improving Precision of Measurements with Least Square Approximation of Pulse Samples with a Second Degree Polynomial

**Figure 4:**
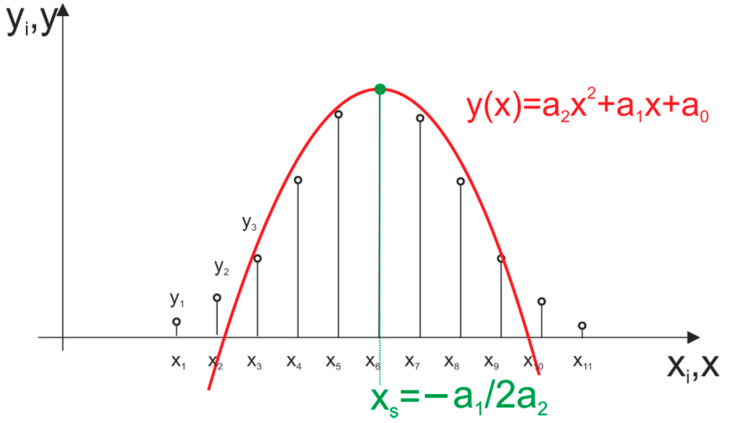
Simple approximation of the discrete echo signal’s waveform with a second degree polynomial (SDPA-S).

The presented method is based on the least square approximation of the shape of a digitally recorded echo signal of a light pulse. The chosen approximating function is a quadratic function (second degree polynomial), as it reassembles the shape of a laser pulse and is easy to calculate. The approximation is performed on the selected subset of *N* recorded samples that is expected to contain the returning echo of a probing pulse. This subset can be selected using constant or adaptive threshold discrimination methods. Approximation is performed using the least square method and returns coefficients of *y*(*x*) quadratic function (parabola) that represent a mathematical model of the received pulse. Distance measurements are based on the determination of the *x_s_* coordinate of the peak of the *y*(*x*) function. Approximation of sampled echo signal with the second degree polynomial is illustrated in Figure 4. Throughout the article, this method is named “second degree polynomial approximation” and abbreviated as SDPA. Two variants of the SDPA algorithm are presented: one is called by the authors a “simple SDPA” and is marked as SDPA-S. The other variant is a method developed by the authors and called a “modified SDPA” (SDPA-M).

In Figure 4, the discrete representation of the received signal is a sequence of samples with their values marked as *y_i_*, for (*i* = 1, 2, …, *N*), obtained by sampling and recording of a signal at x_i_ discrete moments. In the discussed method, it is assumed that the approximation of the real echo signal is a second-degree polynomial function described as:

(1) $$y\left(x\right)=a_{2}x^{2}+a_{1}x+a_{0}$$

where: *y*(*x*) is a function describing the approximating polynomial; *a*_0_, *a*_1_, *a*_2_ are coefficients of the determined polynomial, *x*∈R is the domain of the approximating polynomial.

In order to find the coefficients of the polynomial that best approximates the echo signal, we minimize the mean square error criterion defined as:

(2) $$S=\overset{N}{\underset{i=1}{\sum }}{\left({\hat{y}}_{i}-y_{i}\right)}^{2}\overset{\;}{\to }min$$

where: *N* is the number of selected signal samples, *y_i_* is the value of the sample ‘*i*’, ${\hat{y}}_{i}$ is an estimator of the expected value of the ‘*i*’ sample (the value of the approximating function $\hat{y}\left(x\right)$ calculated for *x_i_*).

When approximating with a second-degree polynomial, the formula (2) can be written as:

(3) $$S\left(a_{0},a_{1},a_{2}\right)=\sum_{i=1}^{N}{\left(a_{2}x_{i}{}^{2}+a_{1}x_{i}+a_{0}-y_{i}\right)}^{2}\overset{\;}{\to }min$$

where: $S\left(a_{0},a_{1},a_{2}\right)$ is the mean square error dependent on coefficients of the polynomial.

The condition of minimizing the mean square error is satisfied when partial derivatives of the function *S*(*a_m_*) (*m* ∈ {0,1,2}) equal zero. Although not necessary to calculate the parabola’s apex position, the formula for *a*_0_ was also shown below for clarity:

(4) $$\frac{\partial S\left(a_{0},a_{1},a_{2}\right)}{\partial a_{0}}=0\to 2\overset{N}{\underset{i=1}{\sum }}\left(a_{0}+a_{1}x_{i}+a_{2}x_{i}{}^{2}-y_{i}\right)=0$$

(5) $$\frac{\partial S\left(a_{0},a_{1},a_{2}\right)}{\partial a_{1}}=0\to 2\overset{N}{\underset{i=1}{\sum }}\left(a_{0}x_{i}+a_{1}x_{i}{}^{2}+a_{2}x_{i}{}^{3}-y_{i}x_{i}\right)=0$$

(6) $$\frac{\partial S\left(a_{0},a_{1},a_{2}\right)}{\partial a_{2}}=0\to 2\overset{N}{\underset{i=1}{\sum }}\left(a_{0}x_{i}{}^{2}+a_{1}x_{i}{}^{3}+a_{2}x_{i}{}^{4}-y_{i}x_{i}{}^{2}\right)=0$$

The above equations can be written in matrix form and solved for coefficients of the approximating polynomial:

(7) $$\left(X^{\prime }X\right)\cdot A-Y_{X}=0$$

(8) $$A={\left(X^{\prime }X\right)}^{-1}\cdot Y_{X}$$

(9) $$A=\frac{{\left(X^{\prime }X\right)}^{D}\cdot Y_{X}}{Det\left(X^{\prime }X\right)}$$

where:

$$\left[\begin{array}{ccc} N & \overset{N}{\underset{1}{\sum }}x_{i} & \overset{N}{\underset{1}{\sum }}x_{i}{}^{2}\\ \overset{N}{\underset{1}{\sum }}x_{i} & \overset{N}{\underset{1}{\sum }}x_{i}{}^{2} & \overset{N}{\underset{1}{\sum }}x_{i}{}^{3}\\ \overset{N}{\underset{1}{\sum }}x_{i}{}^{2} & \overset{N}{\underset{1}{\sum }}x_{i}{}^{3} & \overset{N}{\underset{1}{\sum }}x_{i}{}^{4} \end{array}\right]=X^{\prime }X\cdot \left[\begin{array}{c} a_{0}\\ a_{1}\\ a_{2} \end{array}\right]=A\cdot \left[\begin{array}{c} \overset{N}{\underset{1}{\sum }}y_{i}\\ \overset{N}{\underset{1}{\sum }}y_{i}x_{i}\\ \overset{N}{\underset{1}{\sum }}y_{i}x_{i}{}^{2} \end{array}\right]=Y_{X}$$

$\left(X^{\prime }X\right)$*^D^* is a transposed matrix of algebraic complements [$\left(X^{\prime }X\right)$*_ij_*]*^T^*.

When all coefficients of the polynomial are calculated, the problem of determining the distance comes down to calculation of the position (incidence time) of the peak of the approximated parabola. Signal samples are a set of *N* out of all recorded samples chosen with threshold discriminator and described by a pair of values (*x_i_*, *y_i_*). We find position *x_s_* of signal’s maximum by looking for zero of the first derivative of approximating function:

(10) $$y^{\prime }\left(x_{s}\right)=2a_{2}x_{s}+a_{1}=0\to x_{s}=\frac{-a_{1}}{2a_{2}}$$

Position of the signal’s peak derived from (10) refers to the position in sample’s indexes domain. Transition to the time domain is determined by the relation:

(11) $$t_{STOP}=x_{s}\cdot T=\frac{x_{s}}{f_{s}}$$

where: *t_STOP_* [s] is the time of “STOP” signal generation, *x_s_* is the position of the maximum of the approximated parabola in the domain of sample indices, *T* [s] is the sampling period, *f_s_* [Hz] is the sampling frequency.

The relationship between calculated roundtrip time of the pulse and distance to the target is given by the formula:

(12) $$s=\frac{c_{air}}{2}\cdot (t_{STOP})=\frac{c/n_{air}}{2}\cdot (x_{s}\cdot T)=\frac{c/n_{air}}{2}\cdot \left(\frac{-a_{1}}{2a_{2}}\cdot T\right)$$

where: *s* [m] is the calculated distance, *c* [m/s] is the speed of light in a vacuum, *c_air_* [m/s] is the speed of light in the air, *n_air_* is the index of refraction of air (*n_air_* = 1.0003).

Calculation of distance in a real device requires performance of the following steps:

(1) detection of presence of the echo pulse signal using the chosen detection method; i.e., using constant threshold discrimination,
(2) constitution of the set of *N* samples of echo pulse, which values are greater than the chosen threshold,
(3) calculation of parabola coefficients using solution of formula (9),
(4) calculation of distance using formula (12).

### Analysis of Uncertainty of Presented Method

Variance and standard deviation of polynomial coefficients are calculated from:

(13) $$\sigma_{a0}=\sqrt{Var\left(a_{0}\right)}=\sigma_{y}\sqrt{\frac{\sum_{1}^{N}x_{i}{}^{2}\sum_{1}^{N}x_{i}{}^{4}-{\left(\sum_{1}^{N}x_{i}{}^{3}\right)}^{2}}{Det\left(X^{\prime }X\right)}}$$

(14) $$\sigma_{a1}=\sqrt{Var\left(a_{1}\right)}=\sigma_{y}\sqrt{\frac{N\sum_{1}^{N}x_{i}{}^{4}-{\left(\sum_{1}^{N}x_{i}{}^{2}\right)}^{2}}{Det\left(X^{\prime }X\right)}}$$

(15) $$\sigma_{a2}=\sqrt{Var\left(a_{2}\right)}=\sigma_{y}\sqrt{\frac{N\sum_{1}^{N}x_{i}{}^{2}-{\left(\sum_{1}^{N}x_{i}\right)}^{2}}{Det\left(X^{\prime }X\right)}}$$

The $\sigma_{y}$ coefficient represents the standard deviation of a random noise affecting the value of every sample of a recorded signal; in other words, it can be viewed as an RMS value of the noise in the electronic circuits of the receiver. 

As it was stated, in order to calculate the distance, we are looking for coordinates of the apex of the approximated parabola using Equation (10). It is clear that uncertainties *σ_a_*_1_, *σ_a_*_2_ of coefficients *a*_1_ and *a*_2_ affect the uncertainty of determination of distance *x_s_*. According to the law of propagation of uncertainty, the uncertainty $u_{x_{s}}$ of *x_s_* can be calculated from the formula:

(16) $$u_{x_{s}}=\sqrt{{\left(\frac{-1}{2a_{2}}\sigma_{a_{1}}\right)}^{2}+{\left(\frac{a_{1}}{2{\left(a_{2}\right)}^{2}}\sigma_{a_{2}}\right)}^{2}}$$

where: *u_xs_* is the uncertainty of *x_s_*; *σ_a1_*, *σ_a2_* are the standard deviations of *a_1_* and *a_2_*.

Similarly to the formulas given in (12), the uncertainty calculated using formula (16) is directly proportional to the uncertainty of the distance measurement:

(17) $$u_{s}=\frac{c_{air}\cdot T}{2}\left(u_{x_{s}}\right)=\frac{c_{air}}{2\cdot f_{s}}\left(u_{x_{s}}\right)$$

where: *u_s_* is the uncertainty of the distance measurement.

Formulas (13)–(17) link the distance measurement uncertainty with the properties of recorded echo signals such as Signal-to-Noise ratio and the number of recorded samples based on the relationship between the pulse width and sampling rate. This allows for the estimation of the precision of designed devices while having only basic parameters of used components such as a laser pulse’s peak power and FWHM, clocking the frequency of used analog to digital converters and the expected SNR of the echo signal. In the following section, results of computer simulations that evaluate the effectiveness of formula (17) and the influence of parameters of the signal on the total uncertainty will be shown.

## 4. Simulations

### 4.1. Description of Simulation Assumptions

For the purpose of theoretical analysis, the shape of the incident echo of the probing light pulse is often featured with the Gaussian curve (aka “bell curve”). The slopes of a bell curve are symmetrical in relation to its peak value, and the function extends from negative to positive infinity. Although popular, the approximation of the “shape” of a light pulse with Gaussian function has some drawbacks. The most important is the complexity of a function itself, which requires some computational effort. For the purpose of performed simulations, another function was chosen: a half period of a cosine squared function. It should be noted that actual light pulses generated and processed in laser rangefinders are most often characterized by a lack of symmetry between the rise and fall times of signal’s slopes and the rising slope is usually steeper than the falling one. Still, symmetrical approximation simplifies the analysis and is used in the simulations presented in this article. The presented methods of distance measurements have been implemented and simulated in the MATLAB environment. In order to conduct comparative tests, a waveform imitating the echo signal of a laser pulse was generated. The signal sampling frequency was equal to *f_pr_* = 333 MHz, which means that the time between the acquisition of samples was *T* = 3 ns. Values of samples of the simulated echo pulse were determined using function:

(18) $$y_{i}=f\left(x_{i}\right)=A\cdot cos^{2}\left(\frac{\pi }{2\cdot \lfloor \tau \cdot f_{s}\rfloor }\cdot x_{i}\right)+N\left(0,\sigma_{y}^{2}\right)\;$$

where: 

*x_i_* ∈ 〈⌈*-f_s_∙τ*⌉,⌊*f_s_∙τ*⌋〉 *∩ x_i_* ∈ *Z^+^* is the domain of the *f(x_i_)* function,

*y_i_* is the value of the *i*-th sample of the generated pulse,

*A* is the peak value of generated pulse,

*i* is the ordinal number of the currently generated sample, *i* ∈ {1,2...*n_sig_*},

*n_sig_ = 2*∙⌊*τ∙f_s_*⌋ is the required number of samples included in the generated signal,

⌈ ⌉, ⌊ ⌋ is the “ceiling” and “floor”, round up and down operators.

*N*(0,*σ_y_^2^*) is a random, normally distributed noise with mean value 0 and variance *σ_y_^2^*.

### 4.2. Examination of the Influence of Changes in Signal to Noise Ratio (SNR) and Pulse’s Full Width at Half Maximum (FWHM) on the Total Measurement Error of SDPA Method

In order to validate SDPA algorithms that were presented in Section 3, simulations of distance measurements with various pulse width and signal to noise ratio were performed. Signals were generated using the formula (18) and combined with normally distributed random noise to simulate the real life echo pulses registered by the rangefinder. For each set of signal parameters, an *N* = 1000 simulations were performed, each with a new set of “noise” values added to signal samples. To calculate a distance, the simple polynomial approximation algorithm (11) was used for each dataset selected via fraction discrimination method. From the gathered data, the mean value of a measured distance:

(19) $$\bar{s}=\frac{\sum_{n=1}^{N}s_{n}}{N}$$

and its standard deviation:

(20) $$\sigma_{s}=\sqrt{\frac{\sum_{n=1}^{N}{\left(s_{n}-\bar{s}\right)}^{2}}{N-1}}$$

were calculated. Both results were compared with the values obtained using formulas (15) and (16). Five different scenarios were tested, each with different set of parameters of the signal. The parameters are presented in Table 1. In bold are parameters that are similar to the parameters of the range finder module used in laboratory trials.

**Figure 5:**
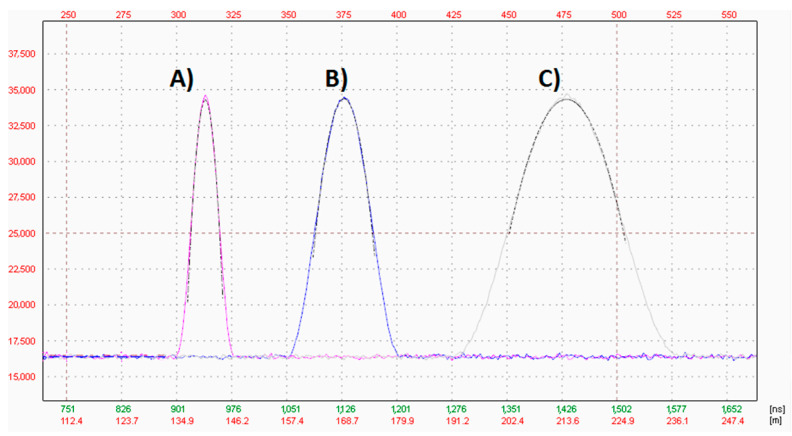
Plots of signals generated for: (A) trial 1, (B) trial 2, (C) trial 3 (see Table 1 for details).

In Figure 5, the three simulated pulses with noise added to achieve the required signal to noise ratio are presented. All signals were used in trials 1, 2 and 3.

The results of simulation trials are presented in Table 2.

The results show that the largest difference between calculated uncertainty u_s_ and *σ_s_* obtained from the trial is no greater than 4%. Thanks to formulas (13)–(18), it is possible to calculate the measurement uncertainty of the SDPA method when having parameters of only a single recorded pulse and without the need to perform numerous trials. By comparison of the results from trial 1 to 3 with trials 4 and 5, it can be noted that the expected relation between measurement uncertainty and SNR was confirmed and uncertainty drops with the increase in SNR. Another conclusion that can be drawn is that uncertainty is directly proportional to the pulse width. Although not tested in this simulations, it is well known that the uncertainty will be inversely proportional to the sampling rate of an ADC.

The main goal for trials 1 to 5 was to validate the correctness of formulas (13)–(18), which bind the relationship between the uncertainty of distance measurements performed with SDPA algorithm with the available quantity *N* of samples of the echo pulse and their relation to electrical noise of the receiver (*σ_y_*). Therefore, for each trial performed, the simulated “distance” of the pulse was constant and known, so that all the recorded samples of the signal were used as input data for the SDPA algorithm. However, in real life, things look different, and the first thing a rangefinder must perform in order to calculate the distance is to find the “location” of the echo of the probing pulse and select the right set of signal samples. This task is usually performed with the constant threshold detection method that is performed either on analog or digital signals fed by the receiver. In the next section of the article, simulations of changing distances are shown so that the influence of the threshold detection and samples selection on measurement error can be analyzed. 

### 4.3. Examination of the Influence of Changes in Distance on the Total Measurement Error—Comparison of Results Given by Different Distance Calculation Algorithms

**Figure 6:**
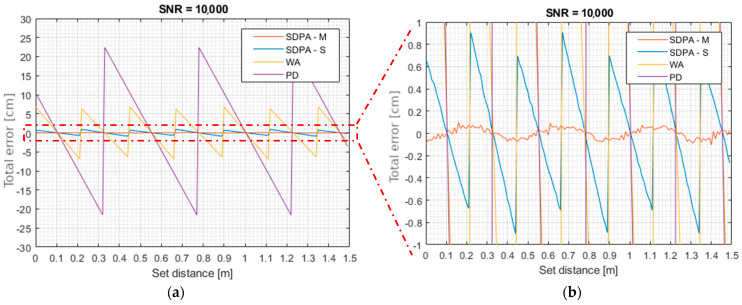
Simulation results. (**a**) Total error Δ*s* of simulated measurements, (**b**) Detailed plot of results of simple and modified approximation algorithms.

In order to examine the influence of sampling and pulse samples selection on the outcome of the distance calculation algorithms, a series of simulation tests was carried out. The set distance was changed within the range of 1.5 m with 1 cm resolution. This ensured that every relationship between the position of the true signal peak and moments of samples collection was covered. The set SNR was very high, so the influence of noise on measurement error could be neglected. Figure 6a shows resulting plots of total distance measurement errors ∆s as a function of set distance. Tested algorithms where based on the peak detection (PD), calculation of weighted average (center of gravity) of the recorded pulse (WA) and least square approximation of the shape of a laser pulse with the second degree polynomial (for both SDPA-S and SDPA-M).

Conducted simulations have shown the strong influence of distribution of signal samples on the results of distance calculations (in relation to the true position of the peak of a signal that marks the correct range). Beside good measurement precision achieved thanks to very high SNR, the results of measurements were inaccurate. It was noticed that for every examined method of distance calculations, errors caused by changes in the peak position and phase of the sampling clock are not random, but cause bias error, which value is related to a time interval Δ*x* between the peak of a true signal and the last sample acquired before it. Changes in measurement bias error were periodic and their plot had a sawtooth shape with a period equal to the period of a sampling clock.

The total error Δ*s* of measurements done with presented algorithms can be seen as a conjunction of two sources of error. One is “responsible” for precision of measurements and can be modeled with normal distribution. For the SDPA algorithm, its standard uncertainty value *u_s_* can be calculated using formulas (13)–(18). The expanded uncertainty (total uncertainty) *U_s_* = 3·*u_s_* can be calculated to model the total measurement error Δ*s*. The second contribution to the total measurement error is caused by the bias error Δ*e_bias_*, which can be modeled with the uniform distribution. In the presented work, its distribution bounds <*a*,*b*> were estimated from the experimental results. The total error of measurements of distance can be noted as:

(21) $$\Delta s=\;\Delta s_{bias}+\;\Delta s_{precission}$$

where:

∆*s_precission_* is the error caused by the noise in the signal,

∆*s_bias_* is the bias error,

The total uncertainty of measurements of a distance can be modeled as expanded uncertainty *U_s_*:

(22) $$\Delta s\approx U_{s}=3\cdot \sqrt{Var\left(e_{precision}\right)+Var\left(e_{bias}\right)}\;$$

where:

*e_precision_* ~ N(0,*u_s_*^2^) is the normally distributed part of uncertainty of a distance measurement caused by the noise,

*e_bias_* ~ U(*a*,*b*) is the systematic error, uniformly distributed within the range <*a*,*b*>,

(23) $$Var\left(e_{bias}\right)=\frac{1}{12}{\left(b-a\right)}^{2}$$

(24) $$Var\left(e_{precision}\right)={\left(u_{s}\right)}^{2}={\left(\sigma_{s}\right)}^{2}$$

So, the total measurement uncertainty *U_s_* can be calculated from:

(25) $$U_{s}=3\cdot \sqrt{{\left(\sigma_{s}\right)}^{2}+\left(\frac{1}{12}{\left(b-a\right)}^{2}\right)}$$

In Table 3, the total uncertainty *Us* of presented algorithms calculated from simulated measurements using formula (25) is shown. Because of the high signal to noise ratio set during simulations, the total error is dominated by the bias error caused by differences in the number of selected samples and their distribution around the true peak of the echo pulse, so the results shown in Table 3 are a close resemblance to a bias error alone.

The bias error of the simplest method (peak detection (PD)) was directly proportional to the value of the signal sampling period. For the period of *T* = 3 ns that was assumed in the simulation, the theoretical, maximum distance measurement error Δ*s* (resulting from the discretization of the signal) equals 22.5 cm. This value corresponds to the one observed in the simulations and estimated in Table 3. Total uncertainty of measurements drops three times thanks to the use of the weighted average algorithm (WA), and twenty-five times thanks to the simple polynomial approximation algorithm. 

**Figure 7:**
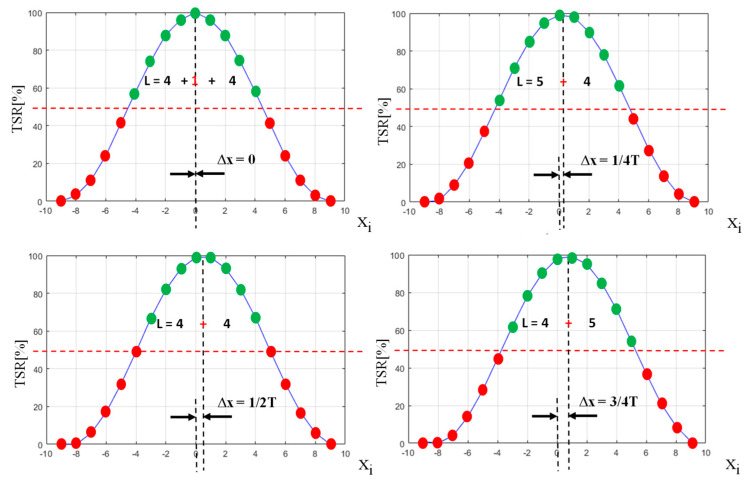
Visualization of results of threshold detection method used on pulses generated with different time delay Δ*x* between their peak and moments of sampling (*TSR*—threshold to signal ratio).

**Figure 8:**
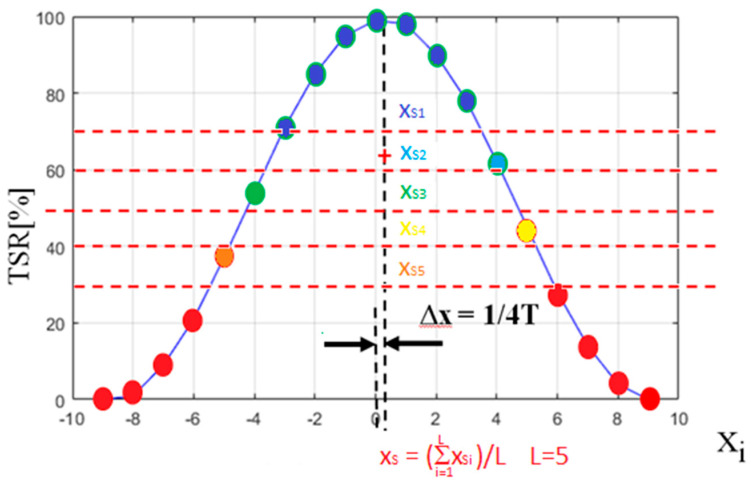
Illustration of the basic concept of calculation of the *x_s_* coordinate of the peak of a pulse using SDPA-M algorithm.

The explanation of the sawtooth bias error *e_bias_* that is caused by the threshold selection of samples is shown in Figure 7. It illustrates four different cases that are significant in terms of changes in the number of samples that exceed the threshold (marked with green). The red dotted line shows the value of a threshold. It is set to 50% of the signal peak value (Threshold to Signal Ratio *TSR* = 0.5). When the sample exceeds the threshold, it is qualified as belonging to the echo pulse. Samples exceeding the threshold constitute a set of input data for the algorithms that calculate the distance, like weighted average (WA) and polynomial approximation (SDPA). In order to reduce the bias error caused by differences in the distribution of samples, efforts were taken to develop an algorithm that counteracts their influence. The modified sample selection algorithm was developed and used with the SDPA approximation algorithm that enabled significant improvement in the measurement accuracy in relation to the simple approximation (SDPA-S) and other methods. The main idea behind this algorithm was to process a single recorded signal multiple times and each time change the value of the threshold used to select the set of input samples for distance calculation algorithms. Doing so produces a set of results that should be averaged to eliminate the bias error. The basic principle of this algorithm is shown in Figure 8.

Presented method was named by the authors the “modified algorithm of pulse shape approximation with second degree polynomial” or simply “modified second degree polynomial approximation” (SDPA-M). Results of measurement error of SDPA-S and SDPA-M obtained during simulations are shown in detail in Figure 6b. From the results shown in Table 3 the reader can see that by using the SDPA-M algorithm, the total uncertainty was reduced almost 200 times in comparison with the PD method. Although the SDPA-M method was invented and tested for use with the polynomial approximation algorithm, it is expected that it will also be useful in eliminating the bias error of the weighted average (WA) algorithm. In the presented example, the threshold value was changed 5 times. Each time, the *x_si_* coordinate of the peak of the approximated parabola was calculated. All five results were averaged to yield *x_s_* value with reduced bias error.

The price to pay for elimination of the bias error is computational effort. It increases significantly when the distance is calculated using the SDPA-M algorithm because the approximation and calculation of the position of the apex has to be done numerous times. For each calculation, also slight modifications in the dataset have to be done. Simple MATLAB tests of the algorithm duration show that pure parabola approximation and calculation of the apex position on a moderately fast PC lasts a few dozen microseconds. The weighted average (WA) algorithm is roughly 20 times faster than SDPA-S. On the other hand, performing multiple calculations with the modified parabola approximation algorithm can take hundreds of microseconds or even a few milliseconds. The computational effort is worth taking when high precision and accuracy are needed and the SNR is sufficiently high, so the error caused by noise does not dominate over the bias error. 

Simulations have shown that the measurement error for input signals with a high signal to noise ratio can be as low as 1 mm; however, in practice, obtaining such high accuracy and precision is extremely difficult due to the increasing influence on the final measurement results of factors like electromagnetic disturbances (both synchronous and asynchronous), the instability of light pulse emitters, background light and atmospheric phenomena. Considering real life measurements, several uncertainty factors resulting from the “outside world” in general should be added to the total uncertainty budget [15]. Those uncertainties can be caused by turbulent atmosphere, the shape of a target [16] or vibrations of a transceiver that cause minor changes in the distance to the target. This uncertainty is usually modeled with normal distribution and the device can reduce it through averaging multiple measurements. It is worth remembering that atmospheric conditions that influence the strength of the echo signal, as well as target reflectivity, are modeled by the influence of the signal to noise ratio (SNR) on the standard uncertainty *u_s_*. 

In the presented article, errors caused by “outside world” factors were not analyzed, as the main goal of the research was to develop a method with precision and accuracy high enough not to dominate the overall uncertainty budget acceptable for devices designed for remote measurements of the speed of vehicles.

## 5. System Tests and Experimental Results

### 5.1. Description of the Test Stand

The aim of the research was to experimentally confirm results obtained during theoretical analyses and simulations, which indicated the possibility of increasing ToF distance measurement accuracy by approximation of the recorded echo pulse signal with a second-degree polynomial.

The laser rangefinder module used in the experiment was designed and built in the Institute of Optoelectronics of the Military University of Technology. It uses a 905 nm pulsed laser that emits 39 ns pulses. Pulse energy equals 0.5 μJ. The rangefinder was connected to a PC with dedicated software that enabled processing of the raw signal data. During tests, the distance to the target and measurement error was determined. Measurements were performed to targets with different, known reflectance.

**Figure 9:**
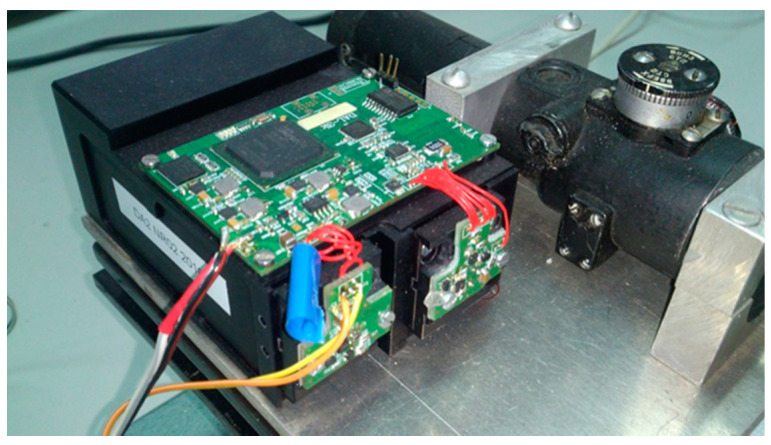
The laser rangefinder module used during experiments.

The measuring stand included:

- a laser rangefinder module with a sighting scope (Figure 9),
- an optical bench with a millimeter scale,
- targets with precisely defined reflectance,
- a computer with an application for system control, data processing and displaying of the results,
- a power supply.

**Figure 10:**
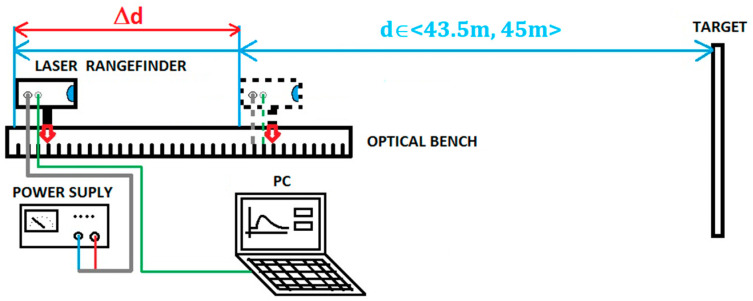
The schematic diagram of the test stand.

The diagram of the stand is shown in Figure 10.

**Figure 11:**
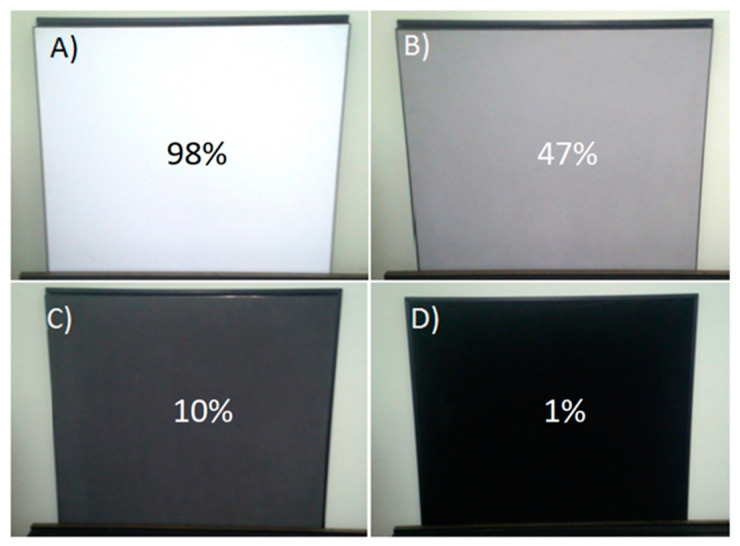
Test targets with calibrated reflectance used in the experiments. Reflectance equals (**A**) 98%, (**B**) 47%, (**C**) 10%, (**D**) 1%.

In order to examine the influence of the changes in the signal to noise ratio (SNR) on the error of distance measurements, it was assumed that the variable parameter (influencing the peak value of received signals) will be the reflectance of targets. Used targets and their corresponding reflectance for the wavelength λ = 905nm are shown in Figure 11.

### 5.2. Methodology of Experiments

The essence of the experiment was to measure differences between set distances (expected value) and measurement results obtained with the laser rangefinder module. PD, WA, SDPA-S and SDPA-M distance measurement algorithms were implemented in the module. The base distance from the target was set as *b* = 43.5 m. The optical bench was positioned along the axis perpendicular to the target. The beginning of the nominal scale on the bench was set at the base distance from the target. Successive measured distances were set by moving the module away from the base distance by a *n* number of Δ*d_step_* steps.

(26) $$\begin{array}{c} set\;distance=base\;distance+displacement=base\;distance+\left(n*set\_step\right)\\ d=b+n\cdot \Delta d_{\mathrm{step}} \end{array}$$

Four measurement cycles were carried out. During each cycle, the target with different reflectance was used. As part of each cycle, a series of 50 measurements was performed for each set distance. A single distance measurement consisted of 1024 laser pulse emissions and recordings of each echo signal. The recorded echo signals were synchronously summed. The purpose of using the coherent addition of signals was to reduce the influence of laser pulses generation jitter on the total measurement error. For each of the tested algorithms, the standard deviation of measurement uncertainty was calculated.

A single step Δ*d_step_* in the change of distance was equal to one centimeter when measurements were taken to a high reflectance target, and two centimeters to targets with low reflectance. The total change in measured distance was Δ*d* = 1.5 m (between 43.5 m and 45 m). The clock frequency of the analog-to-digital converter was *f_pr_* = 333 MHz. This means that the resolution of the recorded digital signal, expressed in the distance domain, was equal to Δ*r* = 0.45 m. The value of the quotient Δ*d*/Δ*r* illustrates how many times during the change of distance by 1.5 m the actual peak of the signal is aligned with the sampling clock. For a given experiment’s assumptions, the real position of the peak moves between three samples, so that every possible difference between the phase of a sampling clock and peak position was covered. Parameters of individual experiments are shown in Table 4.

### 5.3. Results

**Figure 12:**
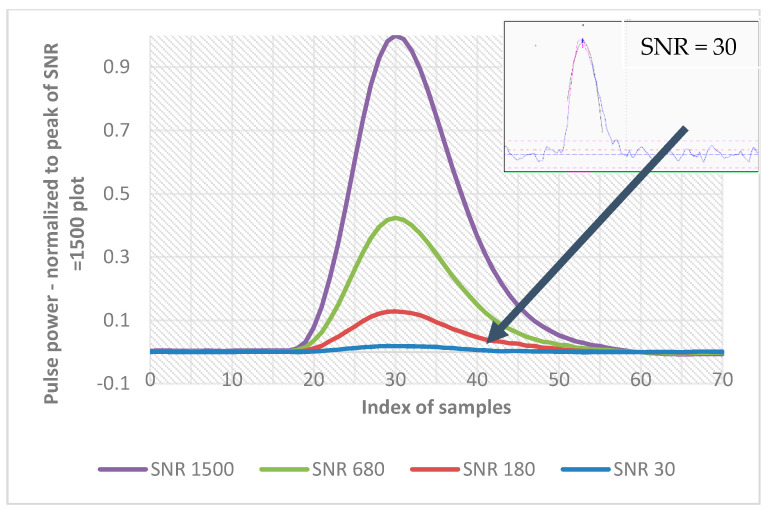
Examples of real life signals registered during measurements to different targets. All signals were normalized to the peak value of the strongest signal. All plots are marked with values of their signal to noise ratios. In the window, the real recorded signal with SNR = 30 is shown.

Figure 12 presents some exemplary plots of the real recorded signal pulses with different SNR. Plots were acquired by making measurements of the same distance, but for different targets (shown in Figure 11). The difference in the signal peak value is clearly visible. 

**Figure 13:**
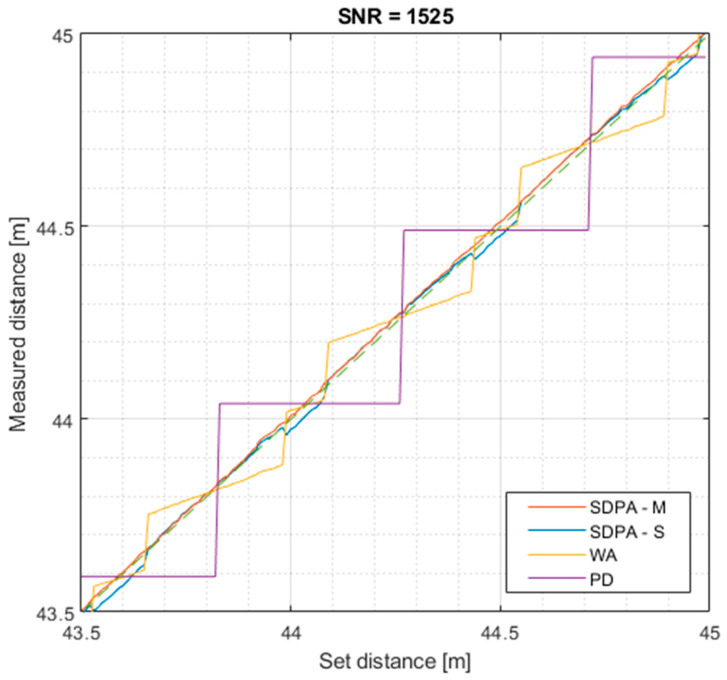
Distance measured with presented algorithms as a function of set distance for SNR = 1525.

Plots in Figure 13 show the distance measurement results obtained in the four tests that were performed. During each test, four distance measurement algorithms were tested that were based on:

- peak detection (PD): finding the time of occurrence of the sample with the maximum value,
- weighted average (WA): calculation of the “center of gravity” of selected signal samples,
- approximation with a second-degree polynomial, simple set of samples (SDPA-S): finding the position of the apex of parabola approximated on the set of echo signal samples selected using the simple threshold discrimination method,
- approximation with a second-degree polynomial, modified set of samples (SDPA-M): finding the average position of the apex of parabola approximated on multiple sets of signal samples selected by the modified sample qualification method.

The reference line that shows ideal measuring results is marked with green color. The purple line marks results obtained with the peak detection (PD) algorithm. The yellow line shows results obtained with weighted average (WA) algorithm. The blue and red lines represent results obtained by algorithms of approximation with the second degree polynomial with, respectively, a simple and modified samples qualification method. Presented plots clearly show that results of the PD method are strongly dependent on the sampling frequency, and the height of the “steps” in Figure 13 represents the distance traveled by the light pulse during one sampling period. The weighted average algorithm is used as a reference method for the SDPA methods. It is less demanding in terms of computational effort and therefore is useful when fast calculations are needed. 

**Figure 14:**
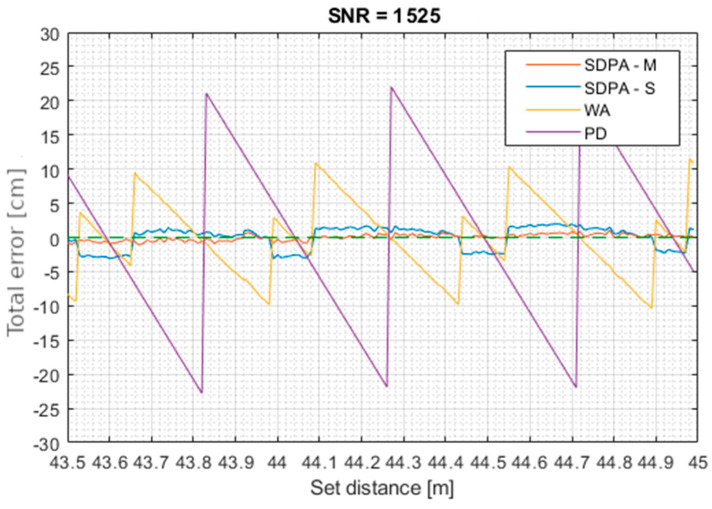
Trial 1. Total error of measurements as a function of set distance for SNR = 1525.

**Figure 15:**
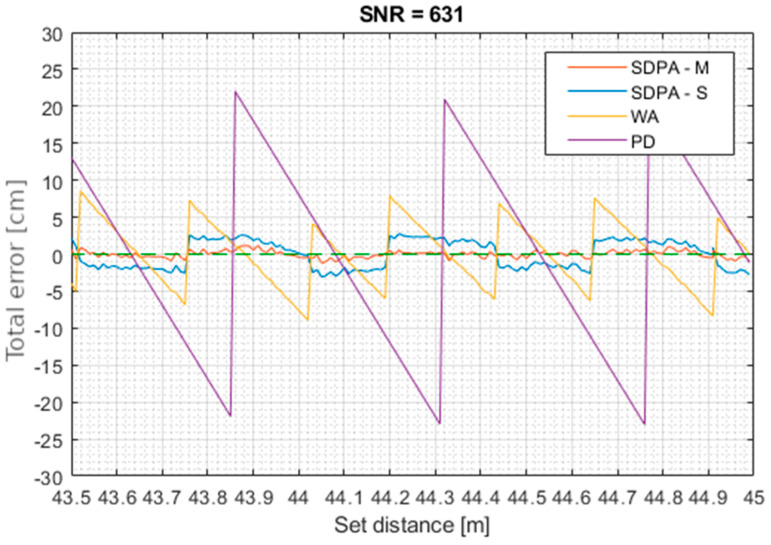
Trial 2. Total error of measurements as a function of set distance for SNR = 631.

**Figure 16:**
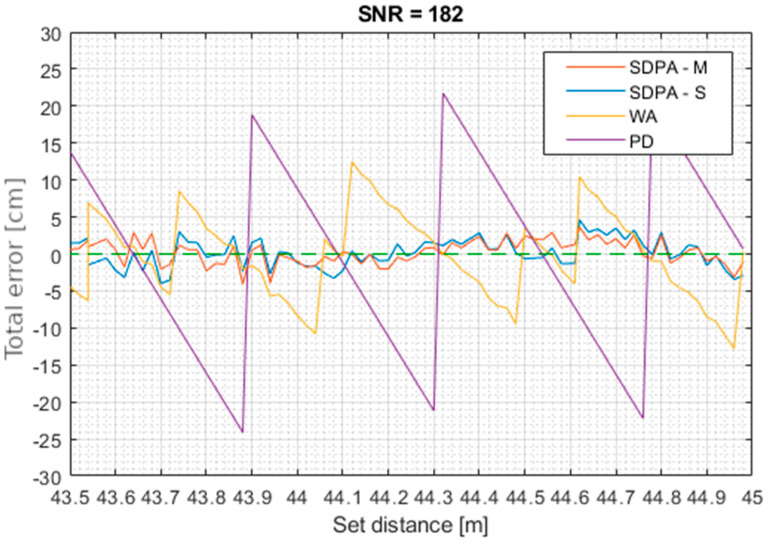
Trial 3. Total error of measurements as a function of set distance for SNR = 182.

**Figure 17:**
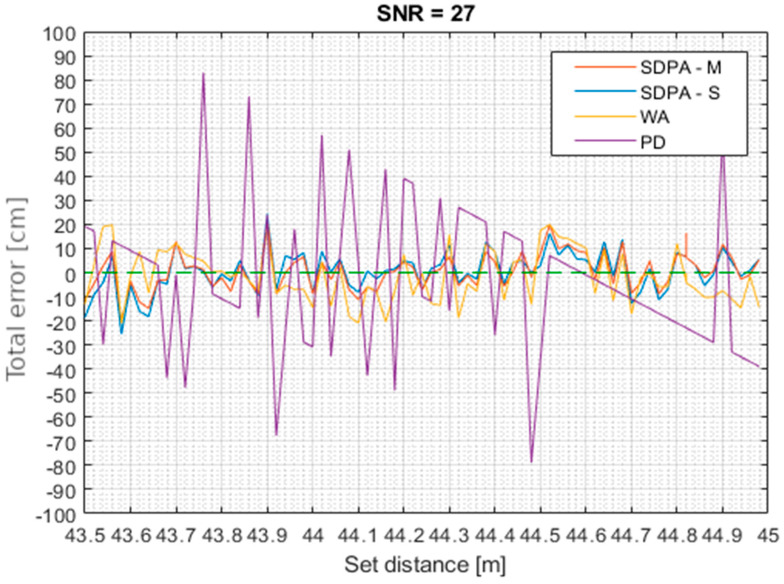
Trial 4. Total error of measurements as a function of set distance for SNR = 27.

Figure 14, Figure 15, Figure 16 and Figure 17 show total error of measurements performed in each trial. Total measurement error *e* was calculated for each gathered data point using formula:

(27) $$\begin{array}{c} total\;error\;of\;measurement=set\;distance-measured\;distance\\ e=d-s \end{array}$$

**Figure 18:**
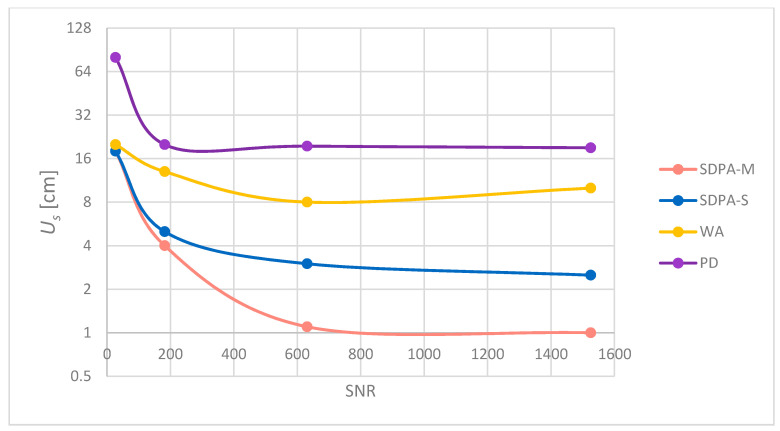
Total uncertainty *U_s_* of each presented algorithm estimated from all four trials.

The measurement uncertainty *U_s_* of each algorithm was estimated using formula (25). Results of calculations are shown in Figure 18 and Table 5.

Analysis of experimental data reveals that the measurement uncertainty of the presented methods consists of the uncertainty caused by the noise and a bias (systematic) uncertainty caused by the chosen method of selection of samples. The modification of the simple SDPA algorithm developed by the authors has significantly reduced this bias error to the level when the total uncertainty of the laboratory measurements (Table 5) is convergent with simulations (Table 2, trial 1 and 4). Differences in the results are thought to be caused by the synchronic disturbances in the real life signal that make the noise distribution differ from the normal distribution and affect the shape of the pulse. Experiments have shown that for signals with high SNR, it is possible to measure the distance with total uncertainty of 1 cm with relatively wide laser pulses (FWHM = 39 ns). All that is thanks to the method of processing of the recorded signals developed by the authors (SDPA-M). The less computation time consuming method SDPA-S also provides superior accuracy over standard detection methods, and is suitable for applications in accurate laser rangefinders and speed guns. The price for high accuracy and precision of presented approximation algorithms is computation time. Obtained experimental results show that for noisy return signals, even the simpler and less demanding methods (SDPA-S, WA and even peak detection) give similar results to “modified polynomial approximation”. It can be noticed that from SNR = 200 down to SNR = 30, the SDPA-S and SDPA-M give similar results. When SNR drops to 30, the uncertainty becomes almost the same for Weighted Average and both SDPA methods. We have further tested the validity of application of the presented algorithms under a deteriorating signal to noise condition down to the SNR value of 6. As was expected, we have observed that the uncertainties of all methods significantly increased and an error caused by the noise dominated. For SNR = 6, the uncertainty of the SDPA-M algorithm has turned out to be the worst among all of the presented methods. According to the obtained results, achieving high measurement precision while using the SDPA-M algorithm is possible when the signal to noise ratio is high (SNR ≥ 180). It is up to the system developer to decide which method to use depending on the expected SNR of returning signals and the dynamic range. The WA method showed some interesting behavior for measurements with SNR = 631 and SNR = 1525. The expected increase in measurement accuracy for higher SNR was not confirmed in the tests. The probable reason for this was the asymmetry (the rising edge is faster than the falling one) of the real received signal pulse that for certain noise levels causes falling edge samples to be more vulnerable to fluctuations when threshold selection of the signal samples is performed. 

## 6. Conclusions

The article presents methods of distance measurements using laser rangefinders with digital acquisition of echo signals and the pulsed Time of Flight principle. The new algorithm that was developed by authors, called “modified second degree polynomial approximation” or SDPA-M, was shown and its measurement uncertainty results were compared with three popular algorithms: peak detection (PD), weighted average (WA) and simple parabola approximation (SDPA-S). Simulations and experimental studies were conducted, showing the accuracy limits of each method in various signal to noise conditions and over various distances. The SDPA-M method has proven superior effectiveness over other methods in low noise conditions (SNR > 180). During simulations, the achieved minimal total error was equal to Δ*s* = 1 mm. During experiments, the estimated uncertainty of the SDPA-M algorithm was calculated as *U_s_* = 1 cm. Two main factors contributing to the total measurement uncertainty were identified: noise that causes decreasing precision, and sampling, which causes bias error (inaccuracy). It has been shown that the SDPA-M algorithm can greatly reduce this bias error. Because all presented algorithms are based on the digitally recorded signals, it is possible to implement digital signal processing methods in order to improve the signal to noise ratio. The most useful algorithms are digital filtration and coherent addition of multiple recordings of signals. Improvement of SNR by coherent addition either increases the device’s maximum range of measurements or allows measurements to be performed to poorly reflecting targets. Thanks to the use of the presented methods and algorithms, it is possible to accurately measure the range to a target over long distances and to any selected point on the target. Simulations and test of the developed SDPA-M algorithm have proven that it meets all given assumptions required for remote laser measurements of speed down to SNR~200. For the price of increasing electronic circuit complexity, its precision can be improved by using analog-to-digital converters with higher sampling rates. Presented second degree polynomial approximation has proven to be sufficient for the given task of allowing laser measurements of the distance with the precision and accuracy of a few centimeters. Such high precision of the generation of STOP signals allows not only for accurate laser distance and speed measurements, but also can be used anywhere there is a need for a laser pulse to carry information about time (i.e., pulsed laser free space optics communications or optical IFF (Identification Friend or Foe) devices). 

## Figures and Tables

**Table 1:** Parameters of generated signals used during trials.

Trial Number	1	2	3	4	5
Sampling frequency *f_pr_* (MHZ)	333				
FWHM *τ* [ns]	**39**	78	156	**39**	156
SNR	**181**			**631**	

**Table 2:** Standard deviations of five trials with *N* = 1000 measurements each. *σ_s_* is calculated from gathered data; u_s_ is calculated using formula (18).

Trial Number	1	2	3	4	5
Standard deviation of the trial, data analysis, *σ_s_* (cm)	1.05	1.17	1.70	0.27	0.46
Calculated standard deviation, *u_s_* (cm)	1.01	1.15	1.65	0.25	0.46
Expanded uncertainty *U_s_* = 3·*u_s_*	**3.03**	3.45	4.95	**0.75**	1.38

**Table 3:** Total estimated uncertainty of selected methods, results of simulations; SNR = 10,000.

SNR	10,000			
Method	SDPA-M	SDPA-S	WA	PD
Total uncertainty *Us* (cm)	0.1	0.8	6	19.5

**Table 4:** Basic characteristics of carried experiments.

Trial Number	1	2	3	4
Target reflectance	98%	47%	10%	1%
Range of distance change	1.5 m			
Distance change step	1 cm		2 cm	
SNR	1525	**631**	**182**	27

**Table 5:** Estimated uncertainty of each method calculated for different SNR conditions.

Calculation Method	Estimated Uncertainty *U_s_* (cm)			
	SNR = 27	SNR = 182	SNR = 631	SNR = 1525
SDPA-M	18	**4**	**1.1**	1
SDPA-S	18	5	3	2.5
WA	20	13	8	10
PD	80	20	19.5	19

## Data Availability

Data is contained within the article.
